# Risk factors and postoperative complications following revision total knee arthroplasty in patients with metabolic syndrome–associated osteoarthritis: a 10-year retrospective analysis using a national inpatient database

**DOI:** 10.3389/fendo.2026.1820941

**Published:** 2026-07-20

**Authors:** Miaolan Yuan, Hao Xie, Yanjie He, Yinyin Qin, Jian Wang

**Affiliations:** 1 Xiaolan Clinical Institute of Shantou University Medical College, Zhongshan, Guangdong, China; 2 Department of General Practice, Xiaolan People's Hospital of ZhongShan (The Fifth People's Hospital of ZhongShan), Zhongshan, Guangdong, China; 3 Division of Orthopaedic Surgery, Department of Orthopaedics, Nanfang Hospital, Southern Medical University, Guangzhou, Guangdong, China

**Keywords:** complications, database, metabolic syndrome associated osteoarthritis, revision total knee arthroplasty, risk factor

## Abstract

**Objective:**

The aim of this study was to examine temporal trends in metabolic syndrome–associated osteoarthritis (MetS-OA) among patients undergoing revision total knee arthroplasty (RTKA) and to identify patient- and clinical-level factors associated with this condition and related outcomes.

**Methods:**

A retrospective cohort analysis was conducted using data from the Nationwide Inpatient Sample from 2010 through 2019. Patient demographics, hospital characteristics, length of stay, total hospitalization charges, in-hospital mortality, comorbid conditions, and perioperative complications were assessed. All analyses incorporated NIS discharge weights, and multivariable logistic regression models were used to assess associations between MetS-OA and clinical outcomes among patients undergoing RTKA.

**Results:**

Among 1,361,454 RTKA hospitalizations identified, 1,330,099 RTKA hospitalizations were included in the analysis. The overall prevalence of MetS-OA was 16.1%, demonstrating a progressive increase from 2011 through 2019. Factors independently associated with MetS-OA included advanced age, male sex, non-White racial background, and the presence of comorbid conditions such as chronic pulmonary disease, depression, and hypothyroidism. Patients with MetS-OA experienced slightly longer hospital stays and incurred higher median total hospitalization charges, exceeding those without MetS-OA by $1,445.50. In-hospital mortality did not differ significantly between groups. MetS-OA was also associated with a higher likelihood of postoperative complications, including acute myocardial infarction, severe malnutrition, acute cerebrovascular disease, postoperative delirium, acute respiratory distress syndrome, prolonged mechanical ventilation, urinary tract infections, acute renal failure, and lower limb nerve injury.

**Conclusion:**

The prevalence of MetS-OA among patients undergoing RTKA has increased over time and is associated with a higher burden of postoperative complications and healthcare utilization. Patient- and hospital-level factors play a substantial role in shaping these outcomes. Targeted preoperative optimization and standardized perioperative management strategies for patients with MetS-OA may mitigate adverse events, enhance postoperative recovery, and reduce overall hospitalization charges.

## 1 Introduction

Osteoarthritis (OA) is a common degenerative joint disorder, affecting an estimated 250 million individuals globally, with a reported prevalence of approximately 60% among adults aged ≥ 65 years (1, 2). From a clinical perspective, OA encompasses several subtypes, including post-traumatic OA, age-related OA, and metabolic syndrome–associated osteoarthritis (MetS-OA) (3). MetS-OA has emerged as a distinct clinical phenotype linked to the broader spectrum of metabolic syndrome (MetS) (4, 5). The association between OA and MetS has been consistently demonstrated across diverse populations and sociocultural contexts, underscoring the systemic nature of this relationship (6).

Amid the global trend of population aging, the prevalence of MetS-OA is rising at an alarming pace, a trend that is anticipated to generate a substantial economic burden. Revision total knee arthroplasty (RTKA) is an important intervention for failed knee arthroplasty and remains a clinically relevant marker of disease burden and healthcare utilization (7–10). Research specifically addressing MetS-OA in this setting remains limited, although the influence of post-traumatic OA on arthroplasty outcomes has been well characterized (11, 12).

This study was undertaken with three primary objectives: (i) to determine the overall prevalence and annual proportion of MetS-OA among patients undergoing RTKA over the past decade; (ii) to assess postoperative adverse outcomes in this population; and (iii) to identify patient- and hospital-level factors associated with MetS-OA and RTKA outcomes. The analysis incorporated patient demographic characteristics, hospital-level factors, length of stay (LOS), total hospitalization charges, in-hospital mortality, comorbidities, and perioperative complications using a national inpatient database.

## 2 Materials and methods

### 2.1 Data source

Data for this study were extracted from the Nationwide Inpatient Sample (NIS), a nationally representative database developed by the Healthcare Cost and Utilization Project and funded by the Agency for Healthcare Research and Quality. The NIS uses a stratified sampling design drawn from more than 1,000 hospitals and captures approximately 20% of all hospitalizations nationwide each year as the largest all-payer inpatient database in the United States (13).

The database contains comprehensive data on patient demographic characteristics, hospital-level variables, LOS, total hospitalization charges, primary payer, in-hospital mortality, and diagnostic and procedural data coded using the International Classification of Diseases, Ninth Revision, Clinical Modification (ICD-9-CM) and the International Classification of Diseases, Tenth Revision, Clinical Modification/Procedure Coding System (ICD-10-CM/PCS). Since the analysis was conducted using publicly available, anonymous data, Institutional Review Board approval was not required.

### 2.2 Cohort selection

The study population consisted of patients aged 18 years and older who underwent revision total knee arthroplasty (RTKA) between 2010 and 2019. Eligible cases were identified from hospital discharge records using ICD-9-CM procedure codes 81.55 and 00.80–00.84 and ICD-10-PCS code groups 0SWC, 0SWD, 0SWT, 0SWU, 0SWV, and 0SWW; complete code specifications are provided in **Supplementary Table 1**.

**Figure 1:**
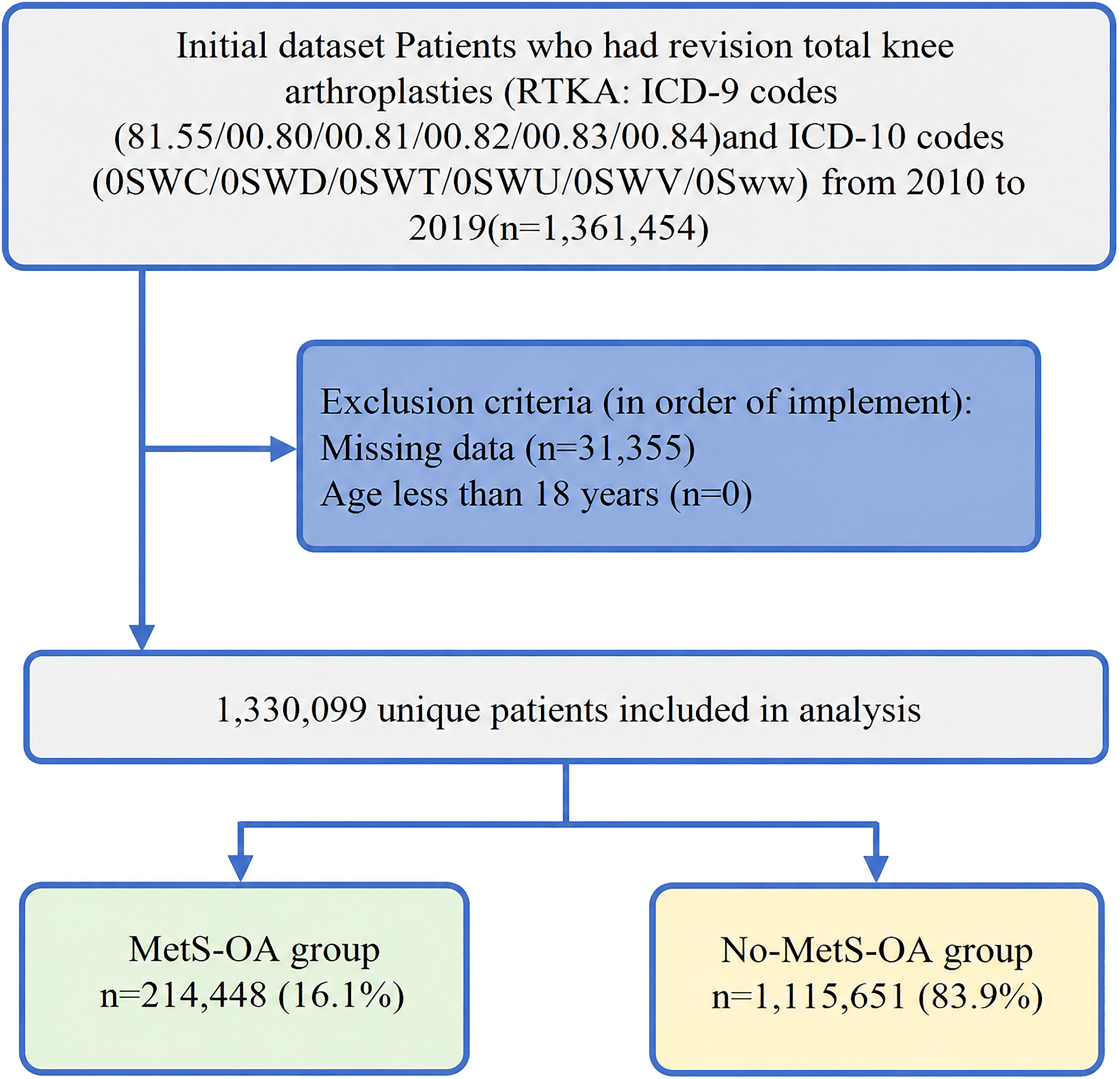
Flow diagram of patient selection from the National Inpatient Sample (NIS) database (2010–2019). The initial cohort included 1,361,454 patients undergoing revision total knee arthroplasty (RTKA), identified by ICD-9-CM procedure codes 81.55 and 00.80–00.84 and ICD-10-PCS code groups 0SWC, 0SWD, 0SWT, 0SWU, 0SWV, and 0SWW (see **Supplementary Table 1**). Exclusion criteria were sequentially applied: missing data (n = 31,355) and age < 18 years (n = 0). The final sample comprised 1,330,099 RTKA hospitalizations, including 214,448 with MetS-OA and 1,115,651 without MetS-OA.

An initial cohort of 1,361,454 patients was identified. Records with missing data on key hospital-level or patient-level variables like age, in-hospital mortality, elective admission status, sex, LOS, insurance type, race, total hospitalization charges, and hospital bed size were subsequently excluded. After these exclusions, the final analytic sample comprised 1,330,099 RTKA hospitalization records, as depicted in **Figure 1**.

The study population was categorized into MetS-OA and non–MetS-OA groups. Operationally, MetS-OA was defined as patients undergoing RTKA who had osteoarthritis together with obesity and at least two metabolic abnormalities captured in the NIS (hypertension, diabetes mellitus, and hyperlipidemia), consistent with a comprehensive definition of metabolic syndrome (14). Patients not meeting this operational definition were classified as non–MetS-OA. The final cohort included 214,448 patients with MetS-OA and 1,115,651 without MetS-OA. Analyses encompassed patient demographic variables, hospital-level characteristics, and clinical outcomes, such as LOS, economic indicators, and in-hospital mortality. Preexisting comorbid conditions and perioperative complications were identified using ICD-9-CM and ICD-10-CM diagnostic codes, as detailed in **Table 1**.

**Table 1:** Variables used in binary logistic regression analysis.

Variable category	Specific variables
Patient demographics	Age (<65 years and ≥65 years), sex (male and female), race (White, Black, Hispanic, Asian or Pacific Islander, Native American and Other)
Hospital characteristics	Type of admission (non-elective, elective), bed size of hospital (small, medium, large), teaching status of hospital (nonteaching, teaching), location of hospital (rural, urban), type of insurance (Medicare, Medicaid, private insurance, self-pay, no charge, other), location of the hospital (northeast, Midwest or north central, south, west)
Comorbidities	AIDS, alcohol abuse, deficiency anemia, rheumatoid diseases, chronic blood loss anemia, congestive heart failure, chronic pulmonary disease, coagulopathy, depression, drug abuse, hypothyroidism, liver disease, fluid and electrolyte disorders, other neurological disorders, paralysis, peripheral vascular disorders, psychoses, pulmonary circulation disorders, renal failure, peptic ulcer disease and valvular disease

AIDS, Acquired immunodeficiency syndrome.

Perioperative complications assessed in this study comprised of acute myocardial infarction, electrolyte imbalances, severe malnutrition, acute cerebrovascular events, pulmonary embolism, gastrointestinal hemorrhage, heart failure, renal insufficiency, pneumonia, acute respiratory distress syndrome (ARDS), prolonged mechanical ventilation, urinary tract infections, acute renal failure, postoperative delirium, blood transfusion requirements, and hemorrhagic complications like seroma and hematoma formation.

### 2.3 Statistical analysis

All statistical analyses were performed using SPSS version 25.0, incorporating NIS discharge weights to account for the complex survey design and to produce nationally representative estimates. Continuous variables were compared using independent t-tests, whereas categorical variables were assessed with chi-squared tests, as presented in **Tables 2** and **3**. Temporal trends in the annual proportion of MetS-OA among RTKA hospitalizations were evaluated using a Cochran-Armitage trend test. Multivariable logistic regression models were used to identify factors associated with MetS-OA and to assess associations between MetS-OA and in-hospital postoperative complications. Effect estimates are reported as odds ratios (ORs), with corresponding 95% confidence intervals (CIs). Considering the large sample size, statistical significance was set at p < 0.05.

**Table 2:** Characteristics and outcomes of RTKA hospitalizations with and without MetS-OA (2010–2019).

Characteristics	Metabolic syndrome associated osteoarthritis (MetS-OA)	No Metabolic syndrome associated osteoarthritis (no MetS-OA)	*p*
Total hospitalizations (n)	214,448	1,115,651	
Total incidence (%)	16.1		
Age (mean±MD)	66.74±8.575	66.21±9.983	<0.05
Age group (%)			
18-44	0.6	1.6	<0.05
45-64	38.0	40.7	
65-74	42.7	36.3	
≥75	18.8	21.4	
Gender (%)			
Male	39.4	37.8	<0.05
Female	60.6	62.2	
Race (%)			
White	73.2	77.4	<0.05
Black	10.2	7.0	
Hispanic	6.2	5.1	
Asian or Pacific Islander	1.5	1.2	
Native American	0.5	0.4	
Other	8.4	8.8	
CCI (%)			
1	33.5	31.8	<0.05
2	18.0	14.5	
≥3	10.9	7.5	
LOS (median, d)	3.0(2-3)	3.0 (2-3)	<0.05
TOTCHG (median, $)	50649.5 (37277.0-71285.8)	49204.0 (36002.0-69695.0)	<0.05
Type of insurance (%)			
Medicare	60.0	55.1	<0.05
Medicaid	4.0	3.8	
Private insurance	32.7	37.3	
Self-pay	0.4	0.5	
No charge	0.0	0.1	
Other	2.9	3.3	
Bed size of hospital (%)			
Small	25.7	26.8	<0.05
Medium	27.8	27.2	
Large	46.5	46.0	
Elective admission (%)	96.0	95.5	<0.05
Type of hospital (teaching %)	57.7	52.8	<0.05
Location of hospital (urban, %)	89.7	88.7	<0.05
Region of hospital (%)			
Northeast	18.0	17.8	<0.05
Midwest or North Central	31.1	26.2	
South	36.6	37.0	
West	14.4	19.0	
Died (%)	0.1	0.0	0.08

LOS, Length of stay; TOTCHG, Total hospitalization charges.

**Table 3:** Relationship between MetS-OA and preoperative comorbidities among RTKA hospitalizations.

Comorbidities	Univariate analysis			Multivariate logistic regression		
	No MetS-OA	MetS-OA	p	OR	95% CI	p
Preoperative comorbidities						
AIDS	763 (0.1%)	142 (0.1%)	0.72	0.65	0.54-0.78	<0.05
Alcohol abuse	10017 (0.9%)	1665 (0.8%)	<0.05	0.73	0.69-0.77	<0.05
Deficiency anemia	75358 (6.8%)	16757 (7.6%)	<0.05	1.06	1.04-1.07	<0.05
Rheumatoid arthritis/collagen vascular diseases	46199 (4.1%)	7394 (3.4%)	<0.05	0.77	0.75-0.78	<0.05
Chronic blood loss anemia	10516 (0.9%)	2153 (1.0%)	<0.05	1.00	0.95-1.05	<0.05
Congestive heart failure	24695 (2.2%)	11035 (5.1%)	<0.05	1.75	1.71-1.80	<0.05
Chronic pulmonary disease	161892 (14.5%)	41164 (19.2%)	<0.05	1.28	1.27-1.30	<0.05
Coagulopathy	20140 (1.8%)	4666 (2.2%)	<0.05	0.99	0.96-1.03	0.72
Depression	148103 (13.3%)	37550 (17.5%)	<0.05	1.37	1.35-1.39	<0.05
Drug abuse	6506 (0.6%)	1244 (0.6%)	0.86	0.87	0.82-0.93	<0.05
Hypothyroidism	180969 (16.2%)	41073 (19.2%)	<0.05	1.22	1.20-1.23	<0.05
Liver disease	13631 (1.2%)	4239 (2.0%)	<0.05	1.43	1.38-1.49	<0.05
Lymphoma	2369 (0.2%)	408 (0.2%)	<0.05	0.77	0.69-0.86	<0.05
Fluid and electrolyte disorders	75509 (6.8%)	205650 (9.6%)	<0.05	1.28	1.26-1.30	<0.05
Other neurological disorders	29780 (2.7%)	5865 (2.7%)	0.09	0.92	0.89-0.94	<0.05
Paralysis	2083 (0.2%)	465 (0.2%)	<0.05	1.01	0.91-1.12	0.81
Peripheral vascular disorders	20531 (1.8%)	6905 (3.2%)	<0.05	1.48	1.43-1.52	<0.05
Psychoses	21937 (2.0%)	5416 (2.5%)	<0.05	1.22	1.18-1.26	<0.05
Pulmonary circulation disorders	9006 (0.8%)	2927 (1.4%)	<0.05	1.21	1.16-1.27	<0.05
Renal failure	46811 (4.2%)	23612 (11.0%)	<0.05	2.46	2.42-2.51	<0.05
Solid tumor without metastasis	5144 (0.5%)	1056 (0.5%)	0.05	0.99	0.93-1.06	0.87
Peptic ulcer disease Excluding bleeding	1542 (0.1%)	390 (0.2%)	<0.05	1.19	1.06-1.33	<0.05
Valvular disease	35499 (3.2%)	8624 (4.0%)	<0.05	1.10	1.07-1.13	<0.05

Data are presented as weighted counts (percentages), representing RTKA hospitalizations. OR, odds ratio; CI, confidence interval.

## 3 Results

### 3.1 Prevalence and temporal trends of MetS-OA in patients undergoing RTKA

**Figure 2:**
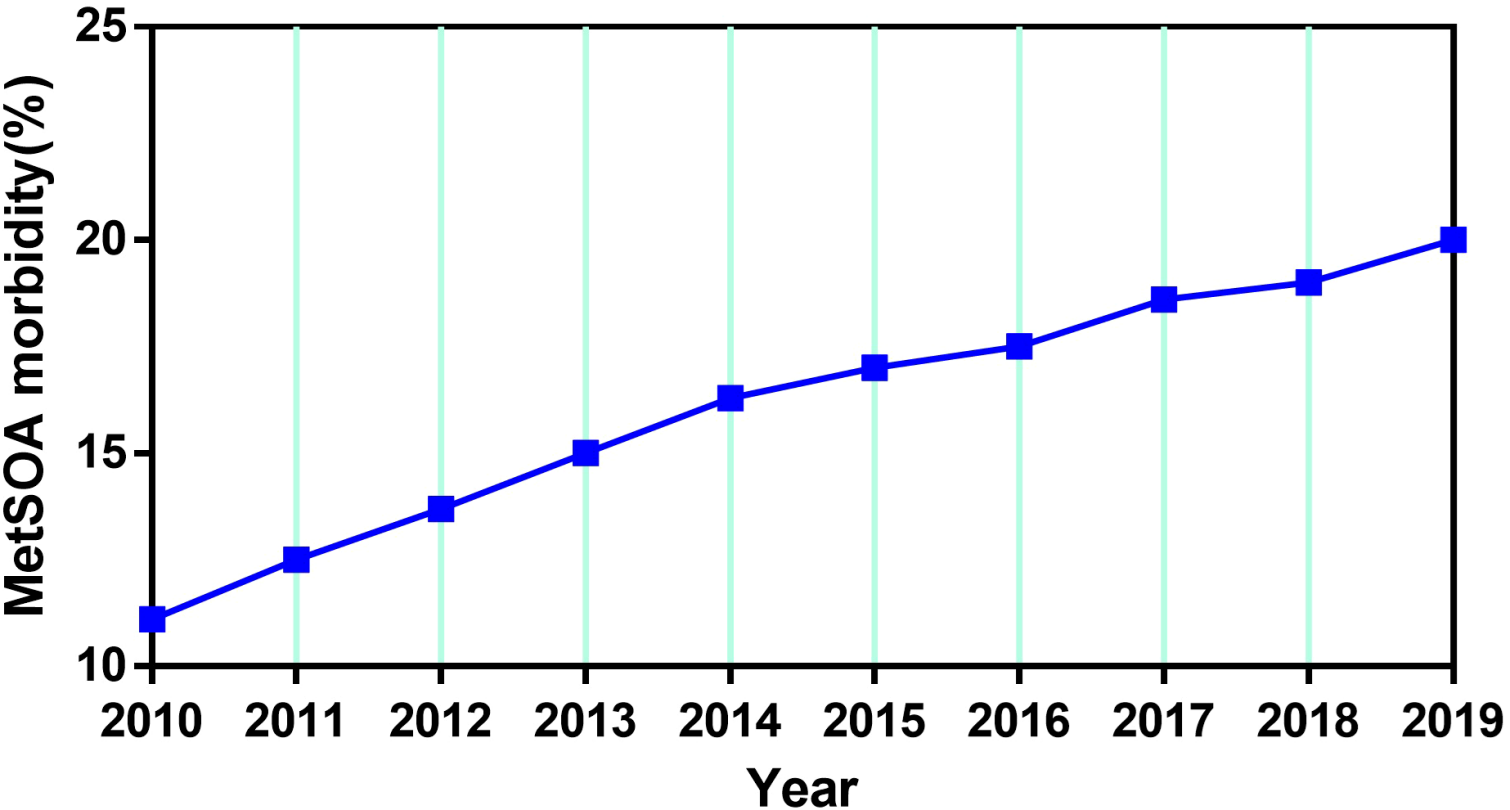
Temporal trends in the prevalence of metabolic syndrome-associated osteoarthritis (MetS-OA) among revision total knee arthroplasty (RTKA) patients (2010–2019). The annual proportion of MetS-OA among RTKA hospitalizations was calculated from the National Inpatient Sample (NIS) database, with values ranging from 10% to 25% over the study period. The line graph illustrates a significant upward trend across the study interval, with the lowest value in 2010 and the highest value in 2019 (p for trend < 0.05).

Analysis of NIS data identified 1,330,099 RTKA hospitalizations in the United States between 2010 and 2019. Of these, 214,448 hospitalization records were classified as having MetS-OA, corresponding to an overall prevalence of 16.1% (**Table 2**). Across the 10-year study period, the annual proportion of patients with MetS-OA undergoing RTKA demonstrated a steady increase, as depicted in **Figure 2**.

### 3.2 Patient demographic characteristics between study groups

Comparative analysis of demographic characteristics indicated that MetS-OA hospitalizations were, on average, one year older than those without MetS-OA (67 years vs. 66 years; *p* < 0.05). The MetS-OA group also included a modestly higher proportion of males (39.4% vs. 37.8%), a difference that reached statistical significance (*p* < 0.05) (**Table 2**).

**Figure 3:**
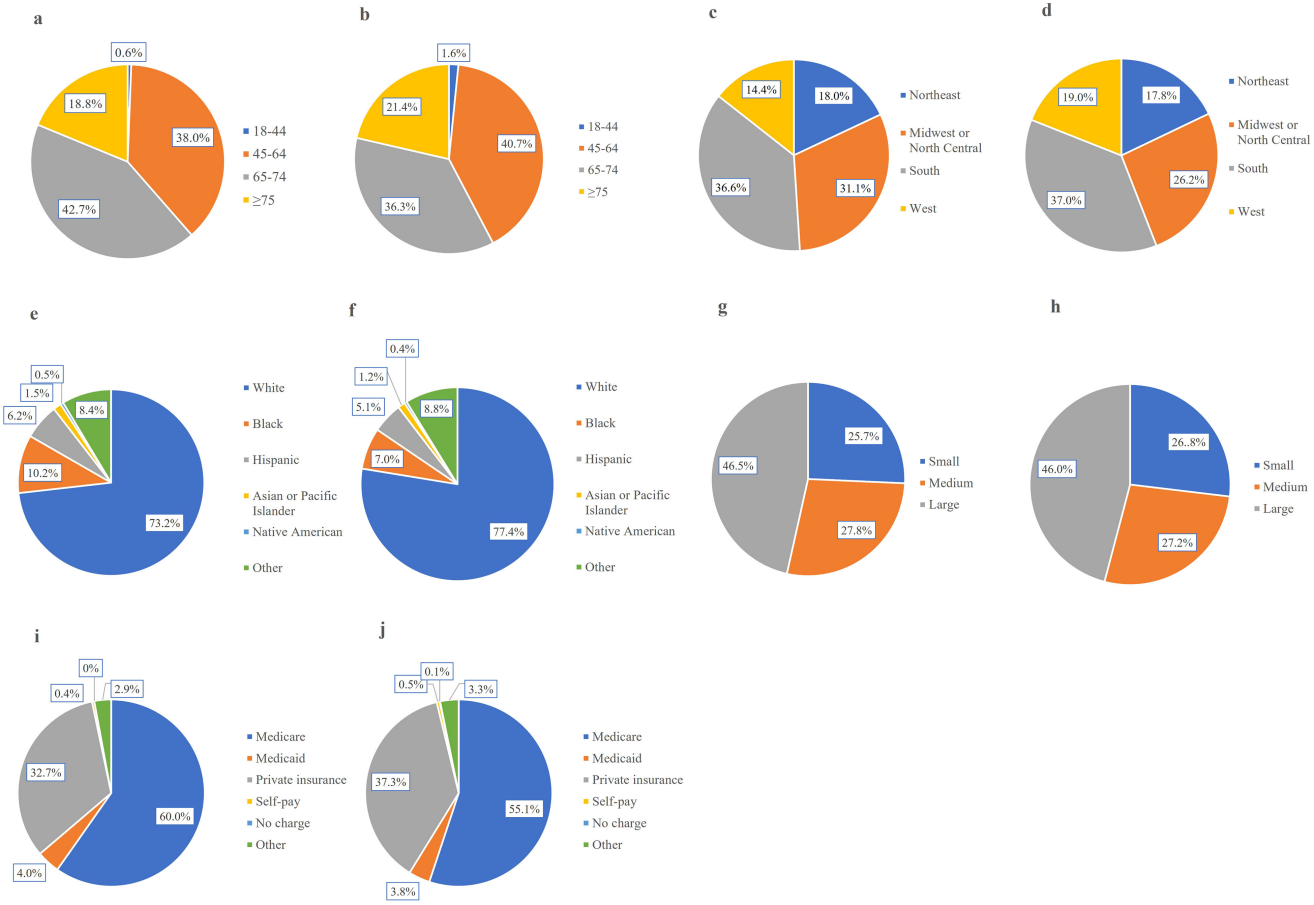
Comparative analysis of demographics and hospital characteristics between MetS-OA and non-MetS-OA patients undergoing revision total knee arthroplasty. **(A)** Age distribution of MetS-OA patients. **(B)** Age distribution of non-MetS-OA patients. **(C)** Geographic distribution of hospitals treating MetS-OA patients. **(D)** Geographic distribution of hospitals treating non-MetS-OA patients. **(E)** Racial/ethnic composition of MetS-OA patients (White: 73.2%, Black: 13.5%, Hispanic: 10.2%, Asian/Pacific Islander: 6.2%, Native American: 8.4%, Other: 0.4%). **(F)** Racial/ethnic composition of non-MetS-OA patients (White: 77.4%, Black: 7.0%, Hispanic: 3.1%, Asian/Pacific Islander: 1.2%, Native American: 0.4%, Other: 10.2%). **(G)** Hospital bed-size categories for MetS-OA patients (Small: 25.7%, Medium: 27.8%, Large: 46.5%). **(H)** Hospital bed-size categories for non-MetS-OA patients (Small: 27.2%, Medium: 26.8%, Large: 46.0%). **(I)** Insurance type distribution for MetS-OA patients. **(J)** Insurance type distribution for non-MetS-OA patients. DATA derived from the National Inpatient Sample (2010–2019). Percentages may not sum to 100% due to rounding or unlisted categories.

Examination of age strata indicated a pronounced difference between groups. Among patients aged 65 to 74 years undergoing RTKA, the prevalence of MetS-OA was 6.4% higher compared with those without the condition (42.7% vs. 36.3%; p < 0.05) (**Table 2**; **Figure 3**). Racial distribution differed significantly between the cohorts, with a lower proportion of White patients observed in the MetS-OA group compared to those without MetS-OA (73.2% vs. 77.4%; p < 0.05) (**Table 2**; **Figures 3E, F**).

### 3.3 Hospital characteristics between study groups

Differences in hospital-related characteristics were observed between MetS-OA and non-MetS-OA hospitalizations. Patients without MetS-OA were slightly less likely to be admitted for elective procedures compared with those diagnosed with MetS-OA (95.5% vs. 96.0%; *p* < 0.05) (**Table 2**). In addition, patients with MetS-OA were more often treated at hospitals with larger bed capacity (46.5% vs. 46.0%; *p* < 0.05) (**Table 2**; **Figures 3G, H**).

Care for patients with MetS-OA was also more commonly provided in urban hospitals (89.7% vs. 88.7%) and in teaching institutions (57.7% vs. 52.8%), with both differences reaching statistical significance (p < 0.05) (**Table 2**). Regional variation was evident, as hospitals located in the Northeast (18.0% vs. 17.8%) and the Midwest/North Central regions (31.1% vs. 26.2%) reported a higher proportion of patients with MetS-OA undergoing RTKA (p < 0.05) (**Table 2**; **Figures 3C, D**).

### 3.4 Preoperative comorbidities associated with MetS-OA during RTKA

**Figure 4:**
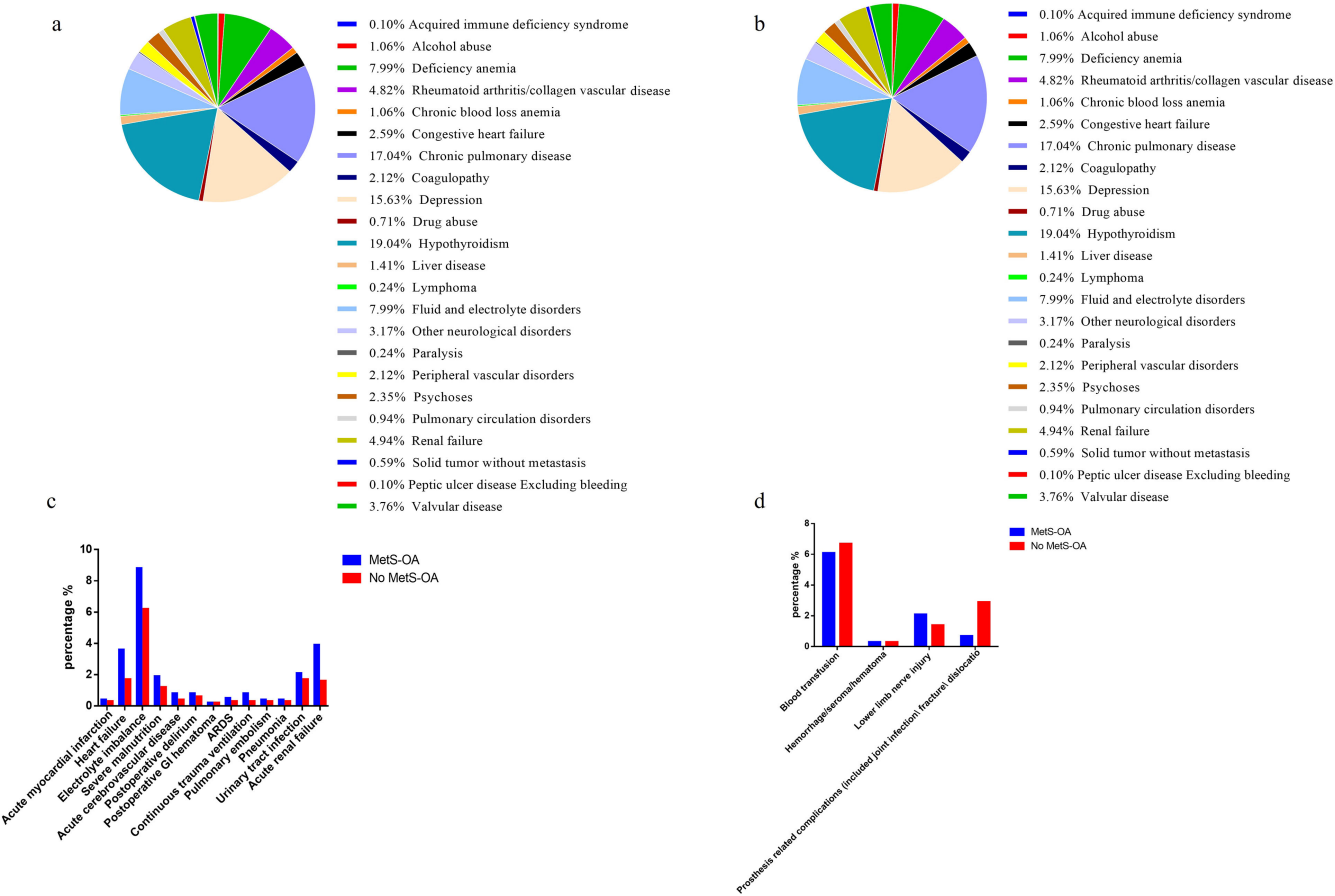
Comparative analysis of preoperative comorbidities between MetS-OA and non-MetS-OA patients undergoing revision total knee arthroplasty. **(A)** Prevalence of preoperative comorbidities in metabolic syndrome-associated osteoarthritis (MetS-OA) patients. Key comorbidities include deficiency anemia (7.6%), congestive heart failure (5.1%), chronic pulmonary disease (19.2%), depression (17.5%), hypothyroidism (19.2%), fluid and electrolyte disorders (9.6%), renal failure (11.0%), and valvular disease (4.0%). **(B)** Prevalence of preoperative comorbidities in non-MetS-OA patients. Data derived from the National Inpatient Sample (2010–2019). Percentages represent the proportion of patients with each comorbidity. **(C)** Medical complications in MetS-OA versus non-MetS-OA patients. **(D)** Surgical complications in MetS-OA versus non-MetS-OA patients. Data derived from the National Inpatient Sample (2010–2019). Bars represent complication rates (%) with 95% confidence intervals. Significant differences between groups are indicated by asterisks (p < 0.05). Adjusted odds ratios (aOR) for complications were calculated using multivariable logistic regression, controlling for age, sex, and comorbidities.

MetS-OA was significantly associated with a range of preexisting comorbid conditions at the time of hospitalization for RTKA. Higher proportions of MetS-OA hospitalizations were observed among those with deficiency anemia (7.6%), congestive heart failure (5.1%), chronic pulmonary disease (19.2%), depression (17.5%), hypothyroidism (19.2%), fluid and electrolyte disorders (9.6%), renal failure (11.0%), and valvular disease (4.0%). Each of these associations reached statistical significance (p < 0.05), as detailed in **Table 3** and depicted in **Figures 4A, B**.

### 3.5 Risk factors associated with MetS-OA in patients undergoing RTKA

Multivariable logistic regression analysis identified several factors independently associated with the presence of MetS-OA among patients undergoing RTKA (**Table 4**). Increasing age was a prominent predictor, with progressively higher odds observed across age categories: 45–64 years (OR = 2.57; 95% CI = 2.42–2.72), 65–74 years (OR = 2.93; 95% CI = 2.76–3.11), and ≥75 years (OR = 1.99; 95% CI = 1.88–2.12), with all associations reaching statistical significance (p < 0.05).

**Table 4:** Risk factors associated with MetS-OA among RTKA hospitalizations.

Variable	Multivariate logistic regression		
	OR	95% CI	p
Age			
18-44	Ref	——	——
45-64	2.57	2.42-2.72	<0.05
65-74	2.93	2.76-3.11	<0.05
≥75	1.99	1.88-2.12	<0.05
Female	0.85	0.84-0.86	<0.05
Race			
White	Ref	——	——
Black	1.53	1.50-1.55	<0.05
Hispanic	1.47	1.44-1.50	<0.05
Asian or Pacific Islander	1.48	1.42-1.54	<0.05
Native American	1.38	1.29-1.48	<0.05
Other	0.97	0.96-0.99	<0.05
Type of insurance			
Medicare	Ref	——	——
Medicaid	0.98	0.96-1.01	0.17
Private insurance	0.87	0.85-0.88	<0.05
Self-pay	0.81	0.75-0.87	<0.05
No charge	0.72	0.58-0.89	<0.05
Other	0.86	0.83-0.88	<0.05
Bed size of hospital			
Small	Ref	——	——
Medium	1.07	1.06-1.09	<0.05
Large	1.06	1.05-1.07	<0.05
Elective admission	1.20	1.17-1.23	<0.05
Teaching hospital	1.15	1.14-1.16	<0.05
Urban hospital	1.03	1.01-1.05	<0.05
Region of hospital			
Northeast	Ref	——	——
Midwest or North Central	1.20	1.19-1.22	<0.05
South	0.96	0.95-0.98	<0.05
West	0.75	0.74-0.76	<0.05

OR, odds ratio; CI, confidence interval.

Also, elective admission status (OR = 1.20; 95% CI = 1.17–1.23; *p* < 0.05), treatment at a teaching hospital (OR = 1.15; 95% CI = 1.14–1.16; *p* < 0.05), and care provided in urban hospitals (OR = 1.03; 95% CI = 1.01–1.05) were each significantly associated with higher odds of MetS-OA (*p* < 0.05).

Conversely, several factors were associated with reduced odds of MetS-OA. Female sex demonstrated a protective association (OR = 0.85; 95% CI = 0.84–0.86; *p* < 0.05), as did geographic location, with hospitals in the Southern (OR = 0.96; 95% CI = 0.95–0.98) and Western regions (OR = 0.75; 95% CI = 0.74–0.76) indicating lower odds compared with other regions (*p* < 0.05) (**Table 4**).

### 3.6 Clinical outcomes associated with MetS-OA in RTKA

MetS-OA hospitalizations exhibited a higher prevalence of multiple comorbid conditions, with 28.9% presenting two or more comorbidities compared with 22.0% among those without MetS-OA (p < 0.05) (**Table 2**). Despite this greater comorbidity burden, in-hospital mortality rates did not differ significantly between the two groups (0.1% vs. 0.0%; p = 0.08) (**Table 2**).

Although the median LOS was similar across groups, patients with MetS-OA had a modestly longer hospitalization distribution overall (both groups 2–3 days; p < 0.05) (**Table 2**). Median total hospitalization charges were significantly higher for patients with MetS-OA, exceeding those of the non–MetS-OA group by $1,445.50 ($50,649.50 vs. $49,204.00; p < 0.05) (**Table 2**).

Differences in payer distribution were noted between groups. Medicare coverage was more common among patients with MetS-OA, exceeding that of the non–MetS-OA group by 4.9% (60.0% vs. 55.1%). Conversely, private insurance coverage was 4.6% lower among MetS-OA patients compared to non–MetS-OA patients (32.7% vs. 37.3%; *p* < 0.05) (**Table 2**; **Figures 3I, J**).

### 3.7 Postoperative complications associated with MetS-OA following RTKA

MetS-OA hospitalizations exhibited higher frequencies of multiple postoperative complications after RTKA. Medical complications observed more commonly in this group included acute myocardial infarction (0.4%), heart failure (3.6%), electrolyte imbalances (8.8%), severe malnutrition (1.9%), acute cerebrovascular disease (0.8%), postoperative delirium (0.8%), ARDS (0.5%), prolonged mechanical ventilation (0.8%), pulmonary embolism (0.4%), pneumonia (0.4%), urinary tract infections (2.1%), and acute renal failure (3.9%).

Along with these medical events, surgical complications were also more common among patients with MetS-OA, particularly lower extremity nerve injury, which occurred in 2.1% of cases. Crude between-group differences are detailed in **Table 5**.

**Table 5:** Relationship between MetS-OA and postoperative complications among RTKA hospitalizations.

Complications	Univariate analysis			Multivariate logistic regression		
	No MetS-OA	MetS-OA	*p*	OR	95% CI	*p*
Medical complications						
Acute myocardial infarction	3776 (0.3%)	960 (0.4%)	<0.05	1.12	1.04-1.21	<0.05
Heart failure	18735 (1.7%)	7751 (3.6%)	<0.05	0.79	0.75-0.83	<0.05
Electrolyte imbalance	69363 (6.2%)	18766 (8.8%)	<0.05	0.93	0.88-0.99	<0.05
Severe malnutrition	13819 (1.2%)	3970 (1.9%)	<0.05	1.36	1.31-1.41	<0.05
Acute cerebrovascular disease	4762 (0.4%)	1642 (0.8%)	<0.05	1.52	1.44-1.62	<0.05
Postoperative delirium	6763 (0.6%)	1640 (0.8%)	<0.05	1.15	1.09-1.22	<0.05
Postoperative GI hematoma	2485 (0.2%)	397 (0.2%)	<0.05	0.72	0.59-0.87	<0.05
ARDS	3284 (0.3%)	1146 (0.5%)	<0.05	1.32	1.23-1.41	<0.05
Mechanical ventilation	3208 (0.3%)	1681 (0.8%)	<0.05	2.09	1.96-2.22	<0.05
Pulmonary embolism	3288 (0.3%)	849 (0.4%)	<0.05	0.97	0.89-1.06	0.55
Pneumonia	3295 (0.3%)	875 (0.4%)	<0.05	1.06	0.98-1.14	0.14
Urinary tract infection	18471 (1.7%)	4531 (2.1%)	<0.05	1.16	1.12-1.20	<0.05
Acute renal failure	17395 (1.6%)	8360 (3.9%)	<0.05	1.69	1.65-1.74	<0.05
Surgical complications						
Blood transfusion	75122 (6.7%)	13073 (6.1%)	<0.05	0.82	0.81-0.84	<0.05
Hemorrhage/seroma/hematoma	3605 (0.3%)	610 (0.3%)	<0.05	0.82	0.75-0.90	<0.05
Lower limb nerve injury	15516 (1.4%)	4570 (2.1%)	<0.05	1.43	1.38-1.47	<0.05
Prosthesis related complications (included joint infection/ fracture/ dislocation)	32037 (2.9%)	1581 (0.7%)	<0.05	0.21	0.20-0.23	<0.05

OR, odds ratio; CI, confidence interval.

Multiple regression analysis demonstrated that patients with MetS-OA were at increased risk for several adverse postoperative outcomes. Elevated odds were observed for acute myocardial infarction (OR = 1.12; 95% CI = 1.04–1.21), severe malnutrition (OR = 1.36; 95% CI = 1.31–1.41), acute cerebrovascular disease (OR = 1.52; 95% CI = 1.44–1.62), postoperative delirium (OR = 1.15; 95% CI = 1.09–1.22), ARDS (OR = 1.32; 95% CI = 1.23–1.41), and prolonged mechanical ventilation (OR = 2.09; 95% CI = 1.96–2.22).

Although the association with pneumonia did not reach statistical significance (OR = 1.06; 95% CI = 0.98–1.14), patients with MetS-OA presented higher odds of developing urinary tract infections (OR = 1.16; 95% CI = 1.12–1.20) and acute renal failure (OR = 1.69; 95% CI = 1.65–1.74) (**Table 5**). In addition, surgical complications were more frequent in this population, with lower extremity nerve injury demonstrating a significantly increased incidence (OR = 1.43; 95% CI = 1.38–1.47) (**Table 5**; **Figures 4C, D**).

## 4 Discussion

As the global population continues to age, the burden of OA has been steadily increasing. In parallel, MetS has been reported to affect more than 25% of the global population in recent decades, with its prevalence continuing to rise (2, 15, 16). These converging trends have contributed to the recognition of MetS-OA as a distinct subtype of the disease.

MetS-OA represents an expanding public health challenge, characterized by a progressively increasing disease burden and growing implications for both clinical management and population health worldwide when compared to other forms of OA. The growing prevalence of this phenotype is closely associated with broader societal changes, including rapid urbanization, higher caloric consumption, rising obesity rates, and more sedentary lifestyles (17).

The well-established relationship between obesity, associated comorbid conditions, and an increased risk of perioperative complications in patients undergoing RTKA and total hip arthroplasty (THA) led the American Association of Hip and Knee Surgeons (AAHKS) to issue a recommendation in 2013 advising that elective arthroplasty procedures be deferred in patients classified as morbidly obese (18).

RTKA is a technically demanding and increasingly common procedure. The annual number of revision total knee arthroplasties in the United States is projected to reach approximately 268,200 by 2030 (7, 10). Despite this growth, the influence of MetS-OA on postoperative outcomes following RTKA, compared with other OA phenotypes, has not been comprehensively examined. Several potentially modifiable factors associated with MetS-OA were identified using 10 years of data from the NIS database.

With respect to demographic characteristics, patients with MetS-OA undergoing RTKA were, on average, one year older than those without MetS-OA (67 years vs. 66 years). This observation differs from prior findings that indicate the prevalence of MetS peaks between 40 and 50 years of age and subsequently stabilizes or declines in older populations. Such differences may be attributable to age-related physiological changes, including reductions in basal metabolic rate, shifts in sex hormone levels, cognitive decline, heightened oxidative stress, and dysregulation of lipid metabolism (19).

In addition, a higher proportion of men with MetS-OA underwent RTKA compared to their counterparts without MetS-OA (39.4% vs. 37.8%). Evidence from a large perspective cohort, the Rotterdam Study, has reported an association between MetS-OA and an increased risk of chronic knee pain in men. In that cohort, abdominal obesity and elevated triglyceride levels were associated with a greater risk of chronic knee pain among men, whereas similar associations were not observed in women (20).

By contrast, findings from studies conducted in the Japanese population have presented sex-specific differences in metabolic profiles, with women exhibiting higher rates of central obesity and reduced high-density lipoprotein cholesterol levels, while men are more likely to experience hypertension and elevated fasting glucose levels (21). Taken together, these observations suggest a potentially greater susceptibility to MetS-OA among men. However, confirmation of this hypothesis requires large-scale, population-specific investigations, particularly given the ethnic heterogeneity and the limited understanding of the biological mechanisms underlying these associations.

The analysis demonstrated clear differences in payer distribution between the two groups. Patients with MetS-OA undergoing RTKA were more frequently insured through Medicare and Medicaid, whereas those without MetS-OA predominantly relied on private insurance or self-payment options (**Table 2**; **Figures 3I, J**). Consistent with these findings, multivariable logistic regression analysis indicated that having a non-Medicare payer was associated with lower odds of MetS-OA among patients undergoing RTKA (**Table 4**).

Patients with MetS-OA were more likely to require hospitalization and to receive care at teaching hospitals. This pattern may reflect the availability of advanced medical technologies and specialized perioperative expertise at these institutions, facilitating more comprehensive management during both the perioperative and postoperative phases (**Table 2**) (22). Also, patients with MetS-OA were more frequently treated at hospitals with a larger bed capacity, which may be related to the greater procedural volume and clinical complexity typically managed at larger centers (**Table 2**).

As anticipated, patients with MetS-OA had similar median LOS but a modestly longer overall hospitalization distribution, together with higher total hospitalization charges (**Table 2**). These differences are likely attributable to the greater perioperative comorbidity burden observed in this population. The higher comorbidity burden may contribute to more resource-intensive perioperative management and greater healthcare utilization (23, 24). Notably, despite these differences, MetS-OA was not associated with increased in-hospital mortality.

A total of 23 comorbid conditions were included in the logistic regression analysis, encompassing factors such as substance use disorders, hypothyroidism, and congestive heart failure (**Table 3**). Several of these conditions emerged as significant risk factors, including deficiency anemia, congestive heart failure, chronic pulmonary disease, depression, hypothyroidism, liver disease, fluid and electrolyte disorders, peripheral vascular disorders, psychotic disorders, pulmonary circulation disorders, renal failure, peptic ulcer disease without bleeding, and valvular heart disease (**Table 3**; **Figure 4**).

Also, selected conditions such as congestive heart failure, depression, psychotic disorders, pulmonary circulation disorders, peptic ulcer disease without bleeding, and valvular heart disease were consistently identified as factors associated with increased risk. These observations are in line with previously published findings, although the biological mechanisms underlying these associations remain incompletely characterized (25, 26). However, recognition of these comorbidities may inform more comprehensive pre-admission health assessments and perioperative risk stratification.

In summary, several factors were identified as significantly associated with MetS-OA among patients undergoing RTKA, including older age, male sex, and non-White racial background. The presence of MetS-OA was associated with a greater burden of preoperative comorbidities, increased utilization of healthcare resources, higher hospitalization charges, and longer LOS. Notably, these associations were not accompanied by an increase in in-hospital mortality.

The findings of this study indicate that patients with MetS-OA undergoing RTKA experienced a higher burden of postoperative complications. These included acute myocardial infarction, severe malnutrition, acute cerebrovascular events, postoperative delirium, ARDS, prolonged mechanical ventilation, urinary tract infections, and acute renal failure. In adjusted analyses, pneumonia did not reach statistical significance. Surgical complications, such as lower limb nerve injury, also occurred more frequently among patients with MetS-OA compared to those without the condition (**Table 5**).

MetS-OA is a complex, multifactorial condition that involves interconnected biochemical and physiological pathways and is frequently associated with cardiovascular disease, type 2 diabetes mellitus, and an increased risk of mortality. Its development has been linked to multiple contributing factors, including insulin resistance, visceral fat accumulation, atherogenic dyslipidemia, endothelial dysfunction, genetic predisposition, hypertension, hypercoagulable state, and chronic psychological stress (14, 27). Among patients undergoing RTKA, MetS-OA is often accompanied by a higher prevalence of fluid and electrolyte disorders and peripheral vascular disorders. These abnormalities may progress to severe and potentially life-threatening complications, such as diabetic ketoacidosis and hyperglycemic hyperosmolar syndrome, which represent the most critical hyperglycemic emergencies in diabetes (28). Peripheral vascular disorders are particularly relevant in this context, as they are commonly associated with both diabetes and hypertension, further complicating perioperative management (29). Hypothyroidism has emerged as a significant risk factor among patients with MetS-OA undergoing RTKA, with evidence suggesting a potential bidirectional relationship between these conditions (30, 31).

Significant evidence indicates that patients with MetS-OA exist in a state of chronic inflammation. The interaction between MetS and OA is mediated by complex biological pathways, including exacerbation of systemic inflammatory responses and increased secretion of adipokines such as adiponectin, leptin, interleukin-6, lipocalin-2, and related mediators that contribute to accelerated chondrocyte aging (32–35). These processes influence both systemic and local immune regulation, promote macrophage polarization, and increase apoptosis of articular chondrocytes (36, 37). Recent studies further emphasize the role of persistent, low-grade systemic inflammation as a crucial factor in the pathogenesis of the disease (38). As a result, MetS-OA is associated with a greater burden of multisystem complications, affecting organs such as the heart, kidneys, and lungs, and is associated with less favorable clinical outcomes. Surgeons should remain attentive to the heightened risk of complications among patients with MetS-OA. Preoperative assessment and optimization strategies should be thoughtfully individualized to address these condition-specific risks (23). In this context, recommendations issued by the AAHKS are consistent with the growing recognition of these challenges and ongoing advances in the perioperative management of this patient population. This study is subject to several limitations inherent to its retrospective design and reliance on large administrative datasets. Most notably, participating hospitals in the NIS database documented patient data solely until discharge. As a result, postoperative complications occurring after discharge were not systematically captured, which may have led to an underestimation of the true incidence of complications associated with MetS-OA. The absence of specific personal data, such as body mass index, hindered a comprehensive analysis of critical risk factors, including operative duration and depth of sedation during post-anesthesia recovery.

Potential coding and reporting biases within administrative data must also be considered, as these factors may have contributed to incomplete identification of patients with MetS-OA. The study offers valuable insights into risk factor profiles among patients with MetS-OA undergoing RTKA despite these constraints. Future studies using prospective study designs are warranted to more accurately delineate risk patterns and temporal trends across different phenotypes of MetS-OA. Further research should also explore the interactions between metabolic components and other preoperative risk factors that may influence postoperative complications following RTKA.

## 5 Conclusion

This analysis demonstrated a rising prevalence of patients with MetS-OA undergoing RTKA between 2010 and 2019, with an overall prevalence of 16.1%. Several factors were identified as being significantly associated with MetS-OA in this population, including older age (≥ 65 years), male sex, treatment at teaching and urban hospitals, and the presence of comorbid conditions such as chronic pulmonary disease and hypothyroidism.

Patients with MetS-OA experienced a higher burden of postoperative complications, longer hospital stays, and increased total hospitalization charges. Addressing these challenges may require targeted preoperative optimization and the development of standardized perioperative management strategies tailored to the specific risks associated with MetS-OA. Such approaches have the potential to reduce complication rates and improve overall patient outcomes following RTKA.

## Data Availability

The original contributions presented in the study are included in the article/**Supplementary Material**. Further inquiries can be directed to the corresponding author.
