# Supplementary material for: Risk factors and postoperative complications following revision total knee arthroplasty in patients with metabolic syndrome–associated osteoarthritis: a 10-year retrospective analysis using a national inpatient database
Source: Front Endocrinol (Lausanne). 2026 Jul 20;17:1820941. doi: 10.3389/fendo.2026.1820941 (PMC13429428; doi:10.3389/fendo.2026.1820941)
Supplement: Supplementary file 1 [file DataSheet1.docx]

**Supplementary Table S1. Procedural codes used to identify revision total knee arthroplasty (RTKA)**

This supplementary table lists the ICD-9-CM and ICD-10-PCS procedure codes/code groups referenced in Section 2.2 of the revised manuscript.

| Coding system | Code / code group | Description |
| --- | --- | --- |
| ICD-9-CM | 81.55 | Revision of knee replacement, not otherwise specified |
| ICD-9-CM | 00.80 | Revision of knee replacement, all components |
| ICD-9-CM | 00.81 | Revision of knee replacement, tibial component |
| ICD-9-CM | 00.82 | Revision of knee replacement, femoral component |
| ICD-9-CM | 00.83 | Revision of knee replacement, patellar component |
| ICD-9-CM | 00.84 | Revision of total knee replacement, tibial insert (liner) |
| ICD-10-PCS | 0SWC | Revision of right knee joint (code group root) |
| ICD-10-PCS | 0SWD | Revision of left knee joint (code group root) |
| ICD-10-PCS | 0SWT | Revision of right femoral surface of knee joint (code group root) |
| ICD-10-PCS | 0SWU | Revision of left femoral surface of knee joint (code group root) |
| ICD-10-PCS | 0SWV | Revision of right tibial surface of knee joint (code group root) |
| ICD-10-PCS | 0SWW | Revision of left tibial surface of knee joint (code group root) |

Note: ICD-10-PCS entries are presented as code groups/root codes because the full procedure code may vary according to specific device and qualifier characters in the discharge record.
